# Serotonergic Input to Orexin Neurons Plays a Role in Maintaining Wakefulness and REM Sleep Architecture

**DOI:** 10.3389/fnins.2018.00892

**Published:** 2018-11-30

**Authors:** Yuki C. Saito, Natsuko Tsujino, Manabu Abe, Maya Yamazaki, Kenji Sakimura, Takeshi Sakurai

**Affiliations:** ^1^Department of Molecular Neuroscience and Integrative Physiology, Faculty of Medicine, Institute of Medical, Pharmaceutical and Health Sciences, Kanazawa University, Kanazawa, Japan; ^2^International Institute for Integrative Sleep Medicine, University of Tsukuba, Tsukuba, Japan; ^3^Department of Cellular Neurobiology, Brain Research Institute, Niigata University, Niigata, Japan; ^4^Department of Animal Model Development, Brain Research Institute, Niigata University, Niigata, Japan; ^5^Faculty of Medicine, University of Tsukuba, Tsukuba, Japan

**Keywords:** sleep, wakefulness, orexin, serotonin, stress

## Abstract

Neurons expressing neuropeptide orexins (hypocretins) in the lateral hypothalamus (LH) and serotonergic neurons in the dorsal raphe nucleus (DR) both play important roles in the regulation of sleep/wakefulness states, and show similar firing patterns across sleep/wakefulness states. Orexin neurons send excitatory projections to serotonergic neurons in the DR, which express both subtypes of orexin receptors (Mieda et al., 2011), while serotonin (5-HT) potently inhibits orexin neurons through activation of 5HT_1A_ receptors (5HT1ARs). In this study, we examined the physiological importance of serotonergic inhibitory regulation of orexin neurons by studying the phenotypes of mice lacking the *5HT1A* receptor gene (*Htr1a*) specifically in orexin neurons (*ox5HT1ARKO* mice). *ox5HT1ARKO* mice exhibited longer NREM sleep time along with decreased wakefulness time in the later phase of the dark period. We also found that restraint stress induced a larger impact on REM sleep architecture in *ox5HT1ARKO* mice than in controls, with a larger delayed increase in REM sleep amount as compared with that in controls, indicating abnormality of REM sleep homeostasis in the mutants. These results suggest that 5HT1ARs in orexin neurons are essential in the regulation of sleep/wakefulness states, and that serotonergic regulation of orexin neurons plays a crucial role in the appropriate control of orexinergic tone to maintain normal sleep/wake architecture.

## Introduction

Orexin A and orexin B, also known as hypocretin-1 and hypocretin-2, respectively, are important regulators of sleep/wakefulness states (Sakurai, 2007, 2014). Orexin/hypocretin-producing neurons (orexin neurons) are located in the lateral hypothalamus (LH) and send axonal projections widely to the entire brain, except the cerebellum, with especially abundant projections to the monoaminergic/cholinergic nuclei in the hypothalamus and brainstem regions (Peyron et al., 1998; Date et al., 1999; Nambu et al., 1999). The actions of orexins are mediated via two G-protein coupled receptors (GPCRs), orexin 1 receptor (OX1R) and orexin 2 receptor (OX2R) (Sakurai et al., 1998). OX1R and OX2R mRNAs exhibit a markedly different and basically complementary distribution, suggesting that these receptors have distinct physiological roles through different neuronal pathways (Sakurai, 2007). The importance of orexins in the maintenance of consolidated sleep/wakefulness states is highlighted by the finding that the sleep disorder narcolepsy is caused by orexin deficiency in several mammalian species, including mice, dogs, and humans (Peyron et al., 1998; Chemelli et al., 1999; Lin et al., 1999; Thannickal et al., 2000). Recent investigations have suggested additional functions of orexins in the regulation of emotions, energy homeostasis, reward systems, drug addiction, and autonomic function (Sakurai, 2014; Soya et al., 2017).

Elucidation of the regulatory mechanisms of orexin neurons is important for understanding the physiological mechanisms of sleep/wakefulness control and the roles of orexin neurons in these functions. Orexin neurons receive innervations from many brain regions, including the limbic system, preoptic area, and monoaminergic neurons (Sakurai et al., 2005; Yoshida et al., 2006; Saito et al., 2018). Among the factors that influence the activity of orexin neurons, serotonin (5-HT) was shown to strongly inhibit most orexin neurons through 5HT_1A_ receptors (5HT1ARs) (Muraki et al., 2004; Tabuchi et al., 2014; Saito et al., 2018). At the same time, GABAergic neurons in the preoptic area that send direct inhibitory projections to orexin neurons are inhibited by 5-HT (Saito et al., 2018), implying that the serotonergic regulation of orexin neurons is complex. A retrograde tracing study of DR 5-HT neurons using rabies virus also showed that orexin neurons send innervations to serotonergic neurons in the DR, suggesting a close relationship between orexin and 5-HT (Pollak Dorocic et al., 2014). However, the function of direct serotonergic regulation of orexin neurons has remained unclear.

In the present study, we generated mice with a selective deletion of the 5HT_1A_ receptor gene (*Htr1a*) specifically in orexin neurons (*Htr1a^f/f^;orexin-Cre* mice, referred to as *ox5HT1ARKO* mice) to study the consequences of deletion of *Htr1a* in orexin neurons in mice to remove the serotonergic regulation of orexin neurons. *ox5HT1ARKO* mice showed shorter wakefulness time during the later phase of the dark period as compared with wild type and control mice. Alteration in the architecture of REM sleep induced by acute restraint stress was more prominent in *ox5HT1ARKO* mice than in controls. These observations suggest that serotonergic regulation of orexin neurons plays an important role in normal maintenance of wakefulness and homeostasis of REM sleep amount.

## Materials and Methods

### Animals

**FIGURE 1:**
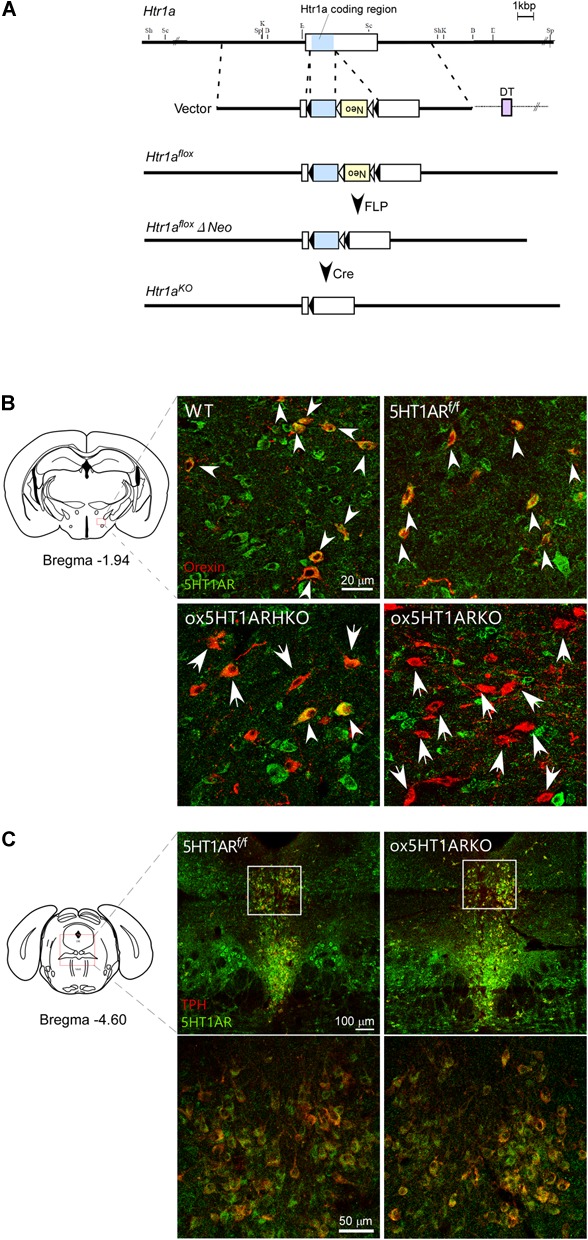
Selective loss of 5HT1AR in orexin neurons in *ox5HT1ARKO* mice. **(A)** Targeting strategy for making *Htr1a^f/f^* mice. **(B)** Matched brain sections from mice with various genotypes were double-stained with anti-orexin (red) and anti-5HT1AR (green) serum. Double-positive cells are shown by white arrowheads. Orexin neurons lacking 5HT1AR expression are shown by arrows. WT, wild type; *5HT1AR^f/f^, Htr1a^f/f^* (*orexin-Cre*-negative); *ox5HT1ARHKO, Htr1a^f/+^* (hetero) mice (*orexin-Cre* transgene-positive); *ox5HT1ARKO, Htr1a^f/f^* mice with *orexin-Cre* transgene. **(C)** Matched brain sections containing the DR from *ox5HT1ARKO* and *5HT1AR^f/f^* were doubly stained with anti-TPH (red) and anti-5HT1AR (green) antibodies, showing similar staining patterns. Left, control (5HT1ARf/f); Right ox5HT1ARKO, lower panels show high power images of corresponding rectangle regions in upper panels.

All experimental procedures involving animals were approved by the Animal Experiment and Use Committee of Kanazawa University (AP-132649) and the University of Tsukuba (18-173), and thus were in accordance with NIH guidelines. *Htr1a^f/f^* mice with inclusion of lox-P sequences in the *htr1a* gene were generated by homologous recombination in C57BL/6N embryonic stem cells and implantation in 8-cell-stage embryos (ICR). A targeting vector was designed to flank the coding region of the *Htr1a* gene by lox-P sites (Figure 1A). Chimeric mice were crossed to C57BL/6J females (Jackson Labs). The *pgk-Neo* cassette was deleted by crossing them with FLP66 mice, which had been backcrossed to C57BL/6J mice more than 10 times. Initially, F1 hybrids from heterozygous × heterozygous mating were generated. We backcrossed them to C57BL/6J mice more than eight times. Genotypes were determined by PCR of mouse tail DNA.

*Orexin-Cre* transgenic mice (Matsuki et al., 2009) were crossed with wild type C57BL6/J mice more than 10 times. *Orexin-Cre* mice were mated with *Htr1a^f/f^* mice, and a breeding colony for producing *orexin-Cre*; *Htr1a^f/f^* was maintained by mating *Ht1ar^f/f^* with *orexin/Cre*; *Ht1ar^f/+^* mice. We used littermates as controls, and all experiments were performed in a C57BL/6J background. We confirmed that wild type mice, *orexin-Cre* transgenic mice, *Ht1ar ^f/+^*;*orexin-Cre*, and *Htr1a^f/f^* (*orexin-Cre*-negative) mice all show the same sleep/wakefulness characteristics (data not shown). In this study, *Htr1a^f/f^*;*orexin-Cre* transgenic mice were used as orexin neuron-selective *5HT1AR* knockout mice (*ox5HT1ARKO*), and *Htr1a^f/+^*;*orexin-Cre* transgenic mice were used as hetero orexin neuron-selective *5HT1AR* knockout mice (*ox5HT1ARHKO*). Their *Htr1a ^f/f^*;*orexin/Cre*-negative littermates (control mice), which show comparable sleep/wakefulness phenotypes to those of wild type C57BL/6J mice, were used as controls. Mice were housed in a temperature- and humidity-controlled, 12 h light/12 h dark room (light on at 9:00), and access water and food *ad libitum*.

### Electrophysiological Study

For electrophysiological analysis, *ox5HT1ARKO* mice were mated with *orexin-eGFP* transgenic mice (Yamanaka et al., 2003) (C57BL/6J background) to obtain *ox5HT1ARKO;orexin-eGFP* mice, which express eGFP as a marker for detecting orexin neurons, and their brain slice preparations were subjected to patch-clamp recordings as previously described (Yamanaka et al., 2003). In brief, brains were extracted and cooled in ice-cold cutting solution consisting of (mM); 87 NaCl, 75 sucrose, 25 NaHCO_3_, 10 D(+)-glucose, 7 MgCl_2_, 2.5 KCl, 1.25 NaH_2_PO_4_, and 0.5 CaCl_2_ bubbled with O_2_ 95% and CO_2_ 5%. Coronal brain slices (250 μm thick) including LHA were prepared with a vibratome (Leica, VT1200S) and incubated for 1 h at room temperature in artificial cerebrospinal fluid (ACSF) containing (mM); 125 NaCl, 26 NaHCO_3_, 10 D(+)-glucose, 2.5 KCl, 2 CaCl_2_, and 1 MgSO_4_ bubbled with O_2_ 95% and CO_2_ 5%. The slices were transferred to a recording chamber (RC-27L, Warner Instrument Corp., Hamden, CT, United States) at room temperature on a fluorescence microscope stage (BX51WI, Olympus, Tokyo, Japan). Neurons that showed GFP fluorescence were used for recordings. The fluorescence microscope was equipped with an infrared camera (C-3077, Hamamatsu Photonics, Hamamatsu, Japan) for infrared differential interference contrast (IR-DIC) imaging and a CCD camera (JK-TU53H, Olympus) for fluorescent imaging. Each image was displayed separately on a monitor. Recordings were carried out with an Axopatch 700B amplifier (Axon Instruments, Foster City, CA, United States) using a borosilicate pipette (GC150-10, Harvard Apparatus, Holliston, MA, United States) prepared by a micropipette puller (P-97, Sutter Instruments, Pangbourne, United Kingdom) and filled with intracellular solution (4–10 MΩ), consisting of (mM): 125 K-gluconate, 5 KCl, 1 MgCl_2_, 10 HEPES, 1.1 EGTA-Na_3_, 5 MgATP, 0.5 Na_2_GTP, pH7.3 with KOH. Osmolarity of the solution was checked by a vapor pressure osmometer (model 5520, Wescor, Logan, UT, United States). The osmolarity of the internal and external solutions was 280–290 and 320–330 mOsm/l, respectively. The liquid junction potential of the patch pipette and perfused extracellular solution was estimated to be -16.2 mV and was applied to the data. The recording pipette was under positive pressure while it was advanced toward individual cells in the slice. Tight seals of 0.5–1.0 GΩ were made by negative pressure. The membrane patch was then ruptured by suction. The series resistance during recording was 10–25 MΩ and was compensated. The reference electrode was an Ag–AgCl pellet immersed in bath solution. Spontaneous action potentials of GFP-expressing neurons were recorded with whole cell modes at 30°C kept by perfusion of preheated ACSF. During recordings, cells were superfused with extracellular solution at a rate of 1.0–2.0 ml/min using a peristaltic pump (K.T. Lab, Japan) at room temperature.

### Histological Analyses

Mice were anesthetized with sodium pentobarbital and perfused transcardially with phosphate-buffered saline (PBS) followed by ice-cold 4% paraformaldehyde. Then, the perfused brains were postfixed overnight in the same fixative buffer and placed in 30% sucrose buffer for 2 days. Cryostat sections (30-μm thick) of the brains were incubated for 1 h in 0.1 M phosphate buffer containing 1% bovine serum albumin and 0.25% Triton-X-100, and incubated overnight at 4°C with the primary antibodies. The primary antibodies used were guinea pig anti-orexin (provided by Prof. Watanabe of Hokkaido University, 1:1000); mouse anti-TPH (Aldrich, T0678, 1:200), and rabbit anti-5HT1AR (Acris, AP06769PU-N, 1:500). Secondary antibodies used were Alexa 488-conjugated donkey anti-rabbit IgG (Invitrogen, A21206, 1:800), Alexa 594-conjugated goat anti-guinea pig IgG (Invitrogen, A11076, 1:800) and Alexa 647-conjugated donkey anti-mouse IgG (Invitrogen, A31571, 1:800). Images were obtained with a fluorescence microscopes (Keyence BZ-9000) or confocal laser scanning microscopes (Leica SP8 and Olympus FV10i).

### Sleep Recordings

Male wild type mice (*n* = 8), *ox5HT1ARKO* mice (*n* = 6), *ox5HT1ARHKO* mice (*n* = 3) and their weight- and age-matched male control littermates: *Htr1a ^f/f^* (*orexin/Cre*-negative) mice (*n* = 9) were implanted with electrodes at 12 weeks of age for EEG/EMG recording, as described previously (Hara et al., 2001). An electrode for EEG and EMG recording was implanted in the skull of each mouse. The three arms of the electrode for EEG recording were placed approximately 2 mm anterior and 2 mm to the right, 2 mm posterior and 2 mm to the right, and 2 mm posterior and 2 mm to the left of the bregma. Stainless steel wires for EMG recording were sutured to the neck muscles of each mouse bilaterally, and each electrode was glued solidly to the skull. After the recovery period, animals were moved to a recording cage placed in an electrically shielded and sound attenuated room. A cable for signal output was connected to the implanted electrode, and animals were allowed to move freely. Mice are singly housed during recordings. Signals were amplified through an amplifier (AB-611J, Nihon Koden, Tokyo) and digitally recorded on a computer using EEG/EMG recording software (Vital recorder, Kissei Comtec). Animals were housed in a 12-h light/dark cycle and allowed to habituate to recording conditions at least 7 days. Each mouse was then recorded for three consecutive 24-h periods, beginning at lights-on at 09:00. Lights were turned off at 21:00. Food and water were replenished at 08:00, and mice were not otherwise disturbed in any way. Averages of each time point on these three recording days were used as raw data, and data from all individual animals used in these studies were used to determine their sleep/wakefulness characteristics.

### Restraint Stress

Stress was applied through restraining mice with a sleeve of nylon-mesh for 30 or 90 min until 21:00, just before the start of the dark period. We folded a nylon mesh sheet (150 mm × 200 mm) in two and hold a mouse in between folds with clips. Mice were released from the restraint, and returned to their cage for sleep-wakefulness recordings.

### Statistical Analysis

Data are presented as mean ± SEM and were analyzed by one-way ANOVA followed by *post hoc* analysis of significance by Fisher’s Protected Least Significant Difference test or paired *t*-test using the GraphPad Prism6 software package (GraphPad Software).

## Results

### Generation of Mice Lacking 5HT1_A_ Receptors Exclusively in Orexin Neurons

To decipher the physiological role of serotonergic regulation of orexin neurons, we examined the phenotype of mice in which orexin neurons selectively lack expression of 5HT1AR, a sole subtype of serotonin receptor in orexin neurons (Muraki et al., 2004). To generate these mice, we first made mice in which the coding region of the gene encoding 5HT1AR (*Htr1a*) is flanked by two *loxP* sites (*Htr1a ^f/f^* mice). Since *Htr1a* has a single exon, we introduced two loxP sites in regions corresponding to the 3′- and 5′-non-coding regions in exon 1 of this gene so that two *loxP* sequences flank the coding region (Figure 1A).

To obtain mice with a deletion of *Htr1a* restricted to orexin-producing neurons, we mated *Htr1a ^f/f^* mice with *orexin-Cre* transgenic mice (Matsuki et al., 2009), in which orexin neurons specifically express Cre recombinase, and obtained *ox5HT1ARKO* mice (*Htr1a ^f/f^;orexin-Cre* mice). Double immunofluorescence studies in wild-type control mice showed that many neurons in the LH possessed 5HT1AR-like immunoreactivity (ir) in their soma and dendrites (Figure 1B). Among these, most orexin-ir cells (orexin-neurons) were positive for 5HT1AR-ir, consistent with our previous observation that all orexin neurons examined showed 5HT1AR-ir and strong hyperpolarization when administered 5-HT (Muraki et al., 2004). Similarly, we also observed that all orexin neurons in *Htr1a^f/f^* mice (Cre-negative) expressed 5HT1AR-ir in their soma and dendrites (Figure 1B). In stark contrast, neurons positive for both 5HT1AR-ir and orexin-ir were barely detectable in the brain of *ox5HT1ARKO* mice, although we observed many 5HT1AR-ir-positive neurons in the LHA that were negative for orexin (Figure 1B). Less than 10% of orexin neurons in *ox5HT1ARKO* mice were double-labeled for orexin and 5HT1AR (Figure 1B). The remaining expression of 5HT1AR in orexin neurons may be due to incomplete penetrance of Cre expression in *orexin-Cre* transgenic mice and/or incomplete deletion of the gene fragment between loxP sites in *ox5HT1ARKO* mice. These histological observations confirmed that in *ox5HT1ARKO* mice, 92.3 ± 8% (*n* = 5) of orexin neurons lack expression of 5HT1AR.

Gross anatomical and histological studies failed to detect any structural abnormalities in the brain of *ox5HT1ARKO* mice. Specifically, the number of orexin neurons in the LH remained normal; the number of immunoreactive cells (located from 0.76 mm to 2.52 mm posterior to the bregma) was 3,424 ± 154 and 3,462 ± 68 for control littermates and *ox5HT1ARKO* mice, respectively (*n* = 8 each).

Figure 1C shows expression of 5HT1ARs in the raphe nuclei in *ox5HT1ARKO* and control mice. Expression of 5HT1AR in these regions in *ox5HT1ARKO* mice was indistinguishable from that in transgene-negative *Htr1a ^f/f^* controls, further supporting that 5HT1AR remained intact in other neurons in *ox5HT1ARKO* mice.

### Electrophysiological Characteristics of Orexin Neurons Lacking 5HT1A Receptors

**FIGURE 2:**
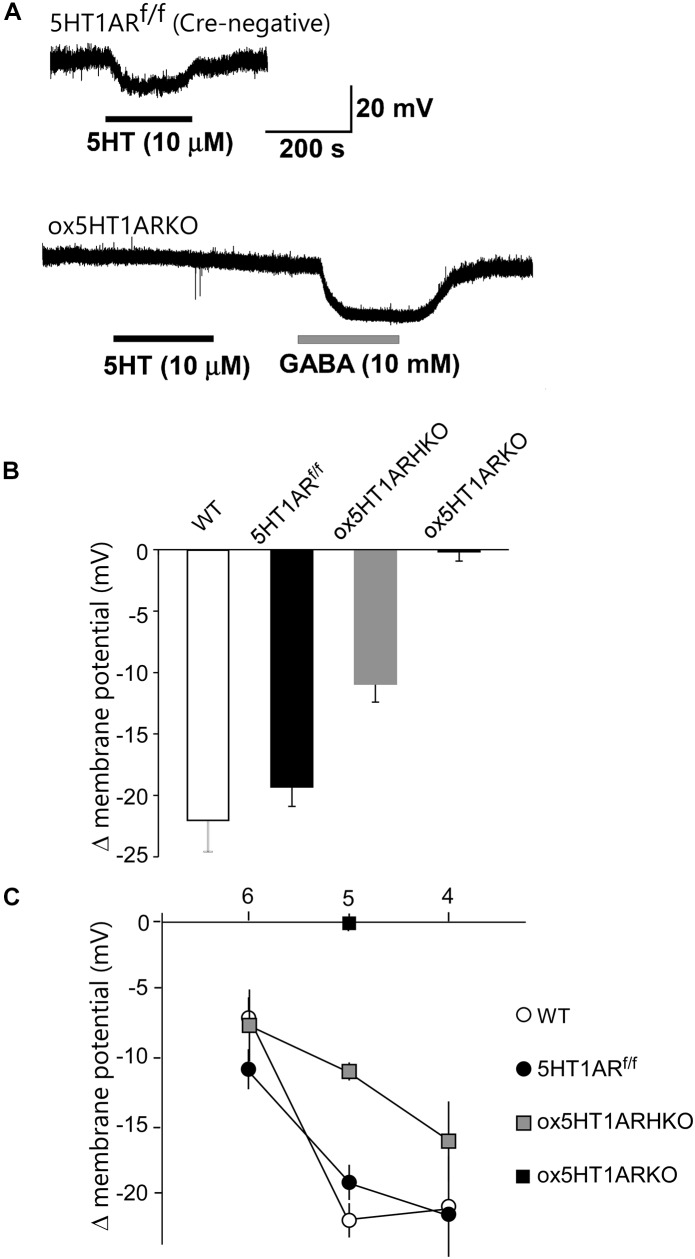
Lack of functional 5HT_1A_ receptors in orexin neurons of *ox5HT1ARKO* mice. (**A**, upper) Representative trace showing the hyperpolarizing effect of 5-HT (10 mM) on orexin neurons in a slice preparation from a control mouse (*5HT1AR^f/f^* mouse without Cre expression) in a current-clamp recording. Drugs were applied during the periods indicated by bars. (**A**, lower) Representative trace showing the effect of 5-HT (10 mM) on orexin neurons in *ox5HT1ARKO* slice. 5-HT does not induce any response, but GABA (10 mM) induces a strong inhibitory response in the same neuron, showing the neuron is viable. **(B)** Hyperpolarization of orexin neurons from mice with various genotypes, induced by 5-HT (10 mM). WT, wild type; *5HT1AR^f/f^, Htr1a^f/f^* (*orexin-Cre*-negative); *ox5HT1ARHKO, Htr1a^f/+^* (hetero) mice (*orexin-Cre* transgene-positive); *ox5HT1ARKO, Htr1a^f/f^* mice with *orexin-Cre* transgene. **(C)** Dose–response curves of 5-HT-induced hyperpolarization of orexin neurons prepared from mice with various genotypes. Horizontal axis show -log dose of 5HT.

We next examined responses of orexin neurons to 5-HT by whole cell patch-clamp recording using brain slice preparations from *ox5HT1ARKO* and control mice (Figure 2). To make identification of orexin neurons easier, we used *ox5HT1ARKO* and control mice possessing the *orexin-eGFP* transgene (Yamanaka et al., 2003). Bath application of 5-HT produced potent, dose-dependent hyperpolarization in all orexin neurons in wild type and control (*Htr1a^f/f^, orexin-Cre* negative) mice, consistent with our previous report (Muraki et al., 2004) (Figures 2A–C). These 5-HT-induced inhibitory effects rapidly reversed upon removal of 5-HT from the external solution (Figure 2A). 5-HT failed to induce hyperpolarization in most orexin neurons in *ox5HT1ARKO* mice (Figure 2B). Very faint hyperpolarization was observed in four out of 19 orexin neurons tested in slices from eight *ox5HT1ARKO* mice, while we observed strong 5-HT-induced hyperpolarization in all neurons examined in wild type and control mice. 5-HT induced a less potent hyperpolarizing effect on orexin neurons of hetero *Htr1a^f/+^*;*orexin-Cre* (*ox5HT1AR*HKO) mice as compared with controls, suggesting that the number of 5HT1ARs in these *ox5HT1AR*HKO mice is lower than that in wild type mice, due to haploinsufficiency (Figure 2C).

Orexin neurons lacking 5HT1AR showed a resting membrane potential similar to that of orexin neurons in control littermates (*ox5HT1ARKO*, -42.2 ± 3.2 mV, *n* = 19; control, -42.3 ± 2.7 mV, *n* = 12, *p* = 0.98).

### *ox5HT1ARKO* Mice Exhibited Decreased Wakefulness Time in Later Phase of Dark Period

**FIGURE 3:**
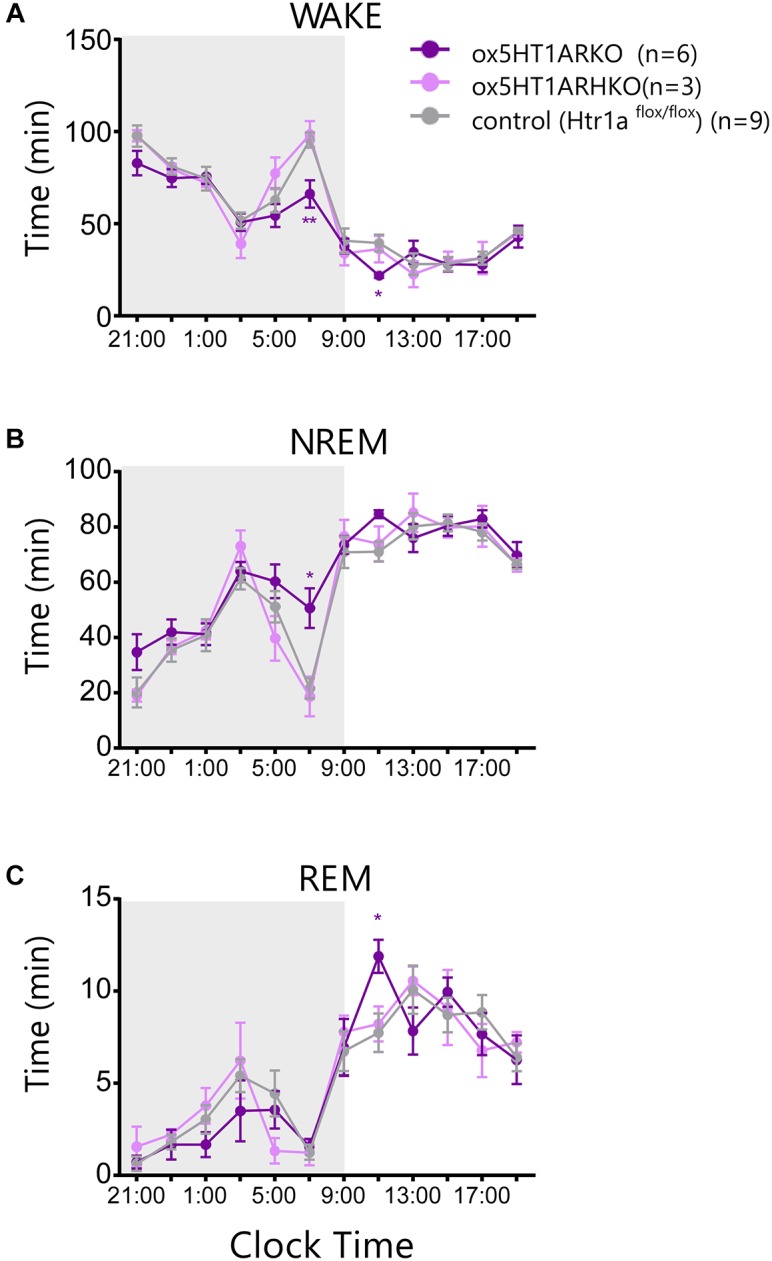
Sleep/wakefulness state patterns of *ox5HT1ARKO, ox5HT1ARHKO* and control littermate mice (*Htr1a^f/f^* mice, negative for Cre) were examined by simultaneous EEG/EMG recordings. Total time of wakefulness (WAKE) **(A)**, NREM sleep **(B)** and REM sleep **(C)** in 2-h periods. ^∗^*p* < 0.05, ^∗∗^*p* < 0.01, significantly different from control mice. Bonferroni test after two-way ANOVA.

Sleep/wakefulness state patterns of *ox5HT1ARKO* mice, *ox5HT1AR*HKO mice, control littermate mice (*Htr1a^f/f^* mice, negative for Cre) and wild type mice were examined by EEG/EMG recording. We found that the total wakefulness time was significantly shorter and NREM sleep was longer in *ox5HT1ARKO* mice than in control mice in the latter half of the dark period (Figure 3 and Table 1). We did not find any difference in episode duration of each state in *ox5HT1ARKO* mice as compared with any other genotypes (Table 1). Neither direct transitions from wakefulness to REM sleep nor cataplexy-like behavioral arrests, which are characteristics of orexin-deficient narcoleptic mice, were observed in *ox5HT1ARKO* mice. The *ox5HT1ARKO* mice show comparable power spectral profiles of EEG with controls during both NREM and REM sleep (Supplementary Figure S1).

**Table 1:** Total time spent in each state (minutes, mean ± SEM), episode duration (seconds, mean ± SEM), REM latency, and interval between successive REM sleep episodes (minutes, mean ± SEM) over 24 h and itemized separately for light and dark periods.

	REM				NREM				WAKE			
	KO	HKO	f/f	WT	KO	HKO	f/f	WT	KO	HKO	f/f	WT
**24 h**					
Total time (min)	63.2 ± 2.9	66.0 ± 3.0	65.1 ± 2.1	57.1 ± 2.4	**^∗^759.9 ± 16.4**	691.1 ± 18.4	678.3 ± 7.4	706.4 ± 18.0	**^∗^616.8** ±**19.0**	682.9 ± 18.8	696.6 ± 6.3	676.7 ± 19.0
Episode duration (min)	1.4 ± 0.1	1.5 ± 0.06	1.4 ± 0.03	1.5 ± 0.06	9.6 ± 1.1	9.6 ± 0.7	8.7 ± 0.5	9.1 ± 0.4	14.4 ± 2.7	18.8 ± 4.0	16.5 ± 1.2	14.2 ± 1.0
REM latency (min)	10.5 ± 1.2	11.5 ± 0.3	10.6 ± 0.7	11.4 ± 0.6						
Inter-REM interval (min)	30.0 ± 3.7	29.2 ± 2.8	27.7 ± 1.1	35.3 ± 2.8						
**Light period**					
Total time (min)	50.6 ± 4.2	49.7 ± 2.2	48.5 ± 3.5	42.2 ± 1.6	467.1 ± 11.4	461.9 ± 14.2	448.0 ± 10.3	436.6 ± 10.2	202.3 ± 13.5	208.4 ± 14.5	223.4 ± 12.8	241.2 ± 11.3
Episode duration (min)	1.5 ± 0.1	1.5 ± 0.06	1.5 ± 0.05	1.6 ± 0.07	9.8 ± 1.2	10.7 ± 0.7	9.3 ± 0.7	10.2 ± 0.7	10.2 ± 1.9	13.3 ± 2.4	10.2 ± 1.1	12.2 ± 1.2
REM latency (min)	10.5 ± 1.2	11.7 ± 0.4	10.6 ± 0.8	11.4 ± 0.7
Inter-REM interval (min)	19.2 ± 2.3	19.5 ± 0.7	19.7 ± 1.3	23.5 ± 1.7
**Dark period**					
Total time (min)	12.7 ± 3.1	16.3 ± 0.8	16.6 ± 2.0	15.0 ± 1.7	**^∗^292.8 ± 12.2**	229.2 ± 4.1	230.3 ± 13.0	269.8 ± 13.3	**^∗^414.6 ± 11.2**	474.4 ± 4.5	473.1 ± 14.4	435.3 ± 14.2
Episode duration (min)	1.2 ± 0.2	1.4 ± 0.1	1.3 ± 0.1	1.5 ± 0.1	9.2 ± 1.1	7.9 ± 0.8	7.8 ± 0.5	8.0 ± 0.5	18.1 ± 3.6	22.2 ± 5.0	14.9 ± 1.7	15.8 ± 1.5
REM latency (min)	10.8 ± 1.1	10.9 ± 0.1	10.6 ± 0.7	11.9 ± 0.8
Inter-REM interval (min)	120.6 ± 50.4	46.7 ± 6.9	49.8 ± 9.5	55.9 ± 7.0

*Significant differences (*p* < 0.05; one-way ANOVA Bonferroni) between each genotype and control mice are indicated with asterisks. KO, *ox5HT1ARKO* (*n* = 6); HKO, ox*5HT1ARHKO* (*n* = 3); *f/f, Htr1a^f/f^* (*n* = 9); WT, wild type (*n* = 8)*.

### 5HT_1A_ Receptors in Orexin Neurons Play an Important Role in Homeostatic Regulation of REM Sleep After Exposure to Acute Stress

**FIGURE 4:**
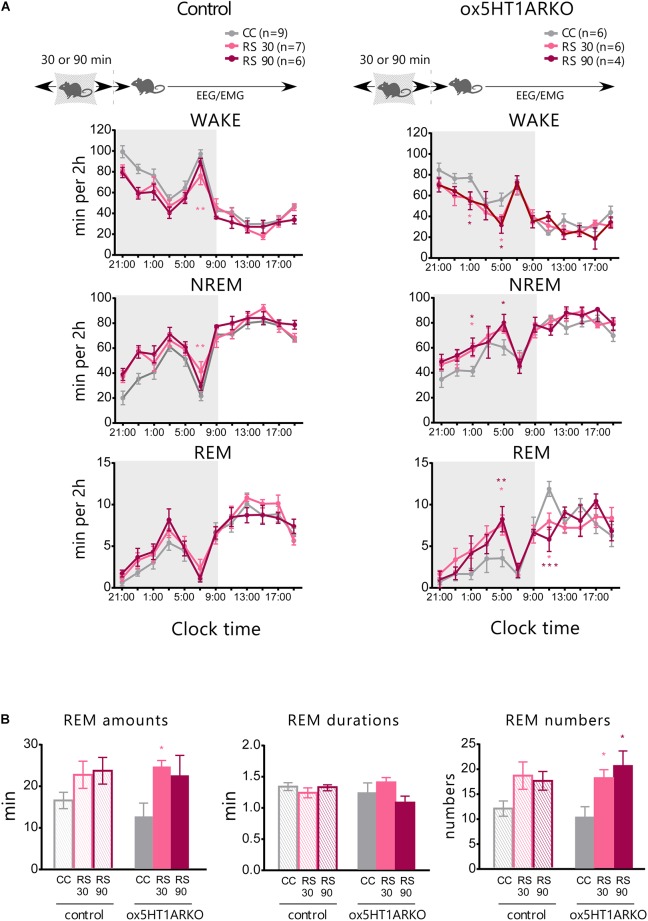
Stress-induced alteration of REM sleep architecture is more prominent in *ox5HT1ARKO* mice as compared with controls (*Htr1a ^f/f^*). **(A)** Effects of 30-min and 90-min mild restraint stress (RS 30, RS 90) in control mice and *ox5HT1ARKO* mice. RS was applied just before the onset of the dark period. **(B)** Amount, duration and episode number of REM sleep during 12-h dark period after application of RS. ^∗^*p* < 0.05, ^∗∗^*p* < 0.01, ^∗∗∗^*p* < 0.001, significantly different from CC (control conditions). Bonferroni test after two-way ANOVA.

Physical stress has been shown to influence the sleep architecture in rats, and especially lead to alteration of the REM sleep structure mediated by serotonergic mechanisms (Rampin et al., 1991). Orexin neurons were shown to be activated by stress (Chang et al., 2007), and orexins potently inhibit REM sleep (Mieda et al., 2011). These observations suggest the possibility that the 5HT^DR^ → orexin pathway plays a role in stress-induced alterations in REM sleep architecture. To evaluate this hypothesis, we applied a restraint stress to *ox5HT1ARKO* and control mice to observe alterations in the sleep architecture. Mice were subjected to mild restraint with nylon mesh for 30 or 90 min just before the onset of the dark period, and subjected to sleep recording. Both control mice and *ox5HT1AKO* mice showed a similar tendency for an increase in NREM sleep amount during the dark period after release from 30- or 90-min restraint stress, which is likely to be attributable to sleep deprivation during stress application. However, the stress did not alter the amount of REM sleep in control mice, while *ox5HT1ARKO* mice responded by an increase in REM sleep amount as compared with the basal condition (Figure 4A). This change was due to an increase in the number of REM sleep episodes, and not to lengthening of REM sleep duration (Figure 4B). *ox5HT1ARKO* mice had a tendency for decreased REM sleep time during the dark period in the basal condition (Figure 3C), but after applying stress, they exhibited a similar amount of REM sleep as compared with control mice. Altogether, these data show that *ox5HT1ARKO* mice exhibited augmented REM sleep enhancement responses to acute physical stress.

## Discussion

Orexin neurons play highly important roles in the maintenance of sleep/wakefulness states (Sakurai, 2007). The regulatory mechanisms of orexin neurons have been examined by many electrophysiological studies *in vitro*, and many factors that can modulate the activity of these neurons have been identified. However, the physiological relevance of the regulatory mechanisms or inputs affecting the activity of orexin neurons remains largely unknown. In our previous study, we examined the phenotypes of mice in which GABA_B_ receptors were selectively removed in orexin neurons (Matsuki et al., 2009). These mice showed highly fragmented sleep/wakefulness states during both the dark and light periods, suggesting the importance of GABA-mediated control of orexin neurons in physiological sleep/wakefulness regulation. Here, we addressed the importance of 5-HT-mediated inhibitory regulation of orexin neurons, since we found 5-HT shows a very potent inhibitory effect on almost all orexin neurons, and 5-HT also plays an important role in the regulation of sleep/wakefulness states. Firing rates of serotonergic neurons are lower during NREM sleep than during wakefulness and lowest during REM sleep, displaying a similar firing pattern to that of orexin neurons across sleep/wakefulness states (Lee et al., 2005; Mileykovskiy et al., 2005; Takahashi et al., 2008). Orexin neurons send excitatory innervation to serotonergic neurons in the raphe nuclei, which abundantly express both OX1R and OX2R (Brown et al., 2002; Liu et al., 2002; Kohlmeier et al., 2013), suggesting that activity of serotonergic neurons is partly regulated by orexin neurons, and this function was shown to be important for inhibition of cataplexy in narcoleptic mice (Mieda et al., 2011; Hasegawa et al., 2017). Direct serotonergic input to orexin neurons might tonically inhibit orexin neurons during wakefulness states, constituting negative feedback regulation. We also confirmed that optogenetic activation of serotonergic fibers in the LH inhibited orexin neurons *in vitro* (Saito et al., 2018). Although chemogenetic activation of 5-HT neurons in the DR showed minimal effects on activity of orexin neurons *in vivo* (Saito et al., 2018), the physiological relevance of direct serotonergic influences on orexin neurons in the regulation of sleep/wakefulness states has been unclear.

Here, we made mice in which orexin neurons selectively lack 5HT1AR, the sole subtype of serotonin receptors expressed in orexin neurons (*ox5HT1ARKO* mice) (Figure 1). *ox5HT1ARKO* mice showed decreased wakefulness time and increased NREM sleep time in the dark period, especially in the later half, as compared with control mice, suggesting that these mice suffer from hypersomnolence. This observation suggests that 5HT1AR-mediated regulation of orexin neurons plays a role in sleep regulation.

Initially, we hypothesized that deletion of serotonergic input to orexin neurons by disrupting the *Htr1a* gene would results in increased wakefulness, because 5-HT strongly inhibits orexin neurons. However, orexin neurons receive complex regulation by the serotonergic system. 5-HT neurons directly inhibit orexin neurons, but indirectly disinhibit these cells through inhibition of GABAergic neurons in the ventrolateral preoptic area (VLPO) that send innervation to orexin neurons (Saito et al., 2018). Therefore, total 5-HT output might have a net excitatory effect on orexin neurons. However, contrary to our initial hypothesis, *ox5HT1ARKO* mice showed increased NREM sleep time in the later half of the dark period as compared with control mice, suggesting that these mice suffer from hypersomnolence (Figure 3). It has been shown that serotonergic tone peaked in the earlier half of the dark period, but decreased in the later half of the dark period in rats (Liu and Borjigin, 2006). This suggests the possibility that decreased serotonergic activity in the later half of the dark period results in a decrease in 5HT1AR-mediated inhibition of orexin neurons, leading to activation of these cells to increase wakefulness. In accordance with this hypothesis, wild type mice show a temporal increase in wakefulness time during the later half of the dark period, which peaks at CT7, but this increase was not observed in *ox5HT1ARKO* mice (Figure 3B). This difference mainly contributes to the decreased amount of wakefulness in *ox5HT1ARKO* mice as compared with control mice. In addition, chronic deficiency of 5HT1AR-mediated inhibition of orexin neurons might result in plastic changes of GABAergic/glutamatergic regulations of orexin neurons. Compensatory increase in GABAergic input and/or decrease in glutamatergic input to orexin neurons might play a role in maintaining wakefulness amount within normal range in the earlier phase of the dark period, but this change might simultaneously decease the activity of these cells in the later half of the dark period, when serotonergic tone is decreased.

The phenotype of *ox5HT1ARKO* mice demonstrates the importance of serotonergic activity within a defined neuronal circuit; in particular, that expression of these 5HT1ARs in orexin neurons is necessary for appropriate maintenance of wakefulness especially during the later half of the dark period. It was shown that overexpression of 5HT1AR in mice rather affected sleep/wakefulness states in the earlier half of the dark period, with fragmentation of sleep/wakefulness (Tabuchi et al., 2014). This also suggests the possibility that the serotonergic influence on orexin neurons is maximal in the earlier half of the dark period, while a decrease in serotonergic influence rather disinhibits orexin neurons to support wakefulness.

We also examined the effect of acute stress on sleep/wakefulness states in *ox5HT1ARKO* mice, because stress might affect serotonergic regulation of orexin neurons. *ox5HT1ARKO* mice showed greater REM sleep rebound after release from restraint, as compared with wild type mice (Figure 4). This result suggests that serotonergic regulation of orexin neurons plays an important role in REM sleep homeostasis. These results suggest the possibility that stress activates orexin neurons, leading to transient activation of 5-HT neurons. Orexin neurons might be disinhibited by decreased serotonergic tone after release from stress, to inhibit REM sleep in wild type mice. REM was gradually increased after the release from the stress, peaking at 8 h after the stress, suggesting that DR serotonergic activity might gradually decrease after the stress over several hours.

This study also provides an insight into the mechanism by which drugs influencing serotonergic tone act on sleep physiology. Compounds such as SSRIs and anti-depressants may increase serotonergic tone in orexin neurons, which could affect sleep/wakefulness states at least partly through dysregulation of orexin neurons. Furthermore, this study suggests that decreased serotonergic influence on orexin neurons might be one of the possible mechanisms of sleep disturbance in patients with depression.

## Author Contributions

YS performed maintenance of mice, sleep analyses, and immunostaining. NT performed electrophysiological studies. MA, MY, and KS generated 5HT1A floxed mice. TS designed the experiments, wrote the manuscript, and performed analyses.

## Conflict of Interest Statement

The authors declare that the research was conducted in the absence of any commercial or financial relationships that could be construed as a potential conflict of interest.

## Supplementary Material

FIGURE S1 EEG power density of NREM sleep **(A)** and REM sleep **(B)** are shown as the mean percentage of total EEG power ± SEM in control littermate (*n* = 7) and *ox5HT1ARKO* mice (*n* = 6) for 0.25-Hz frequency bins between 0.25 and 20 Hz. The delta range (0.75–4 Hz) is indicated by the black bar and the theta range (6–9 Hz) is indicated by the gray bar. Bonferroni test after two-wayANOVA.
