# Supplementary figures and images for: Serotonergic Input to Orexin Neurons Plays a Role in Maintaining Wakefulness and REM Sleep Architecture
Source: Front Neurosci. 2018 Nov 30;12:892. doi: 10.3389/fnins.2018.00892 (PMC6284013; doi:10.3389/fnins.2018.00892)

A

NREM

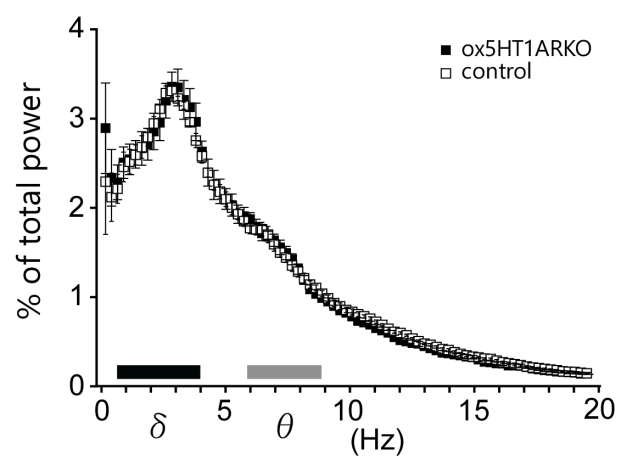

B

REM

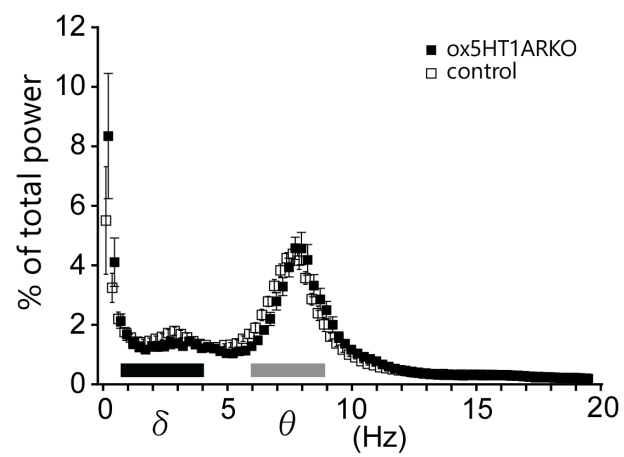

Supplement: FIGURE S1 — EEG power density of NREM sleep (A) and REM sleep (B) are shown as the mean percentage of total EEG power ± SEM in control littermate (n = 7) and ox5HT1ARKO mice (n = 6) for 0.25-Hz frequency bins between 0.25 and 20 Hz. The delta range (0.75–4 Hz) is indicated by the black bar and the theta range (6–9 Hz) is indicated by the gray bar. Bonferroni test after two-wayANOVA. [file Data_Sheet_1.PDF]
